# RsTTG1, a WD40 Protein, Interacts with the bHLH Transcription Factor RsTT8 to Regulate Anthocyanin and Proanthocyanidin Biosynthesis in *Raphanus sativus*

**DOI:** 10.3390/ijms231911973

**Published:** 2022-10-09

**Authors:** Sun-Hyung Lim, Da-Hye Kim, Jong-Yeol Lee

**Affiliations:** 1 Division of Horticultural Biotechnology, School of Biotechnology, Hankyong National University, Anseong 17579, Korea; 2 Research Institute of International Technology and Information, Hankyong National University, Anseong 17579, Korea; 3 National Institute of Agricultural Sciences, Rural Development Administration, Jeonju 54874, Korea

**Keywords:** anthocyanin biosynthesis, MBW complex, proanthocyanidin biosynthesis, RsTTG1, WD40 protein

## Abstract

MBW complexes, consisting of MYB, basic helix–loop–helix (bHLH), and WD40 proteins, regulate multiple traits in plants, including anthocyanin and proanthocyanidin (PA) biosynthesis and the determination of epidermal cell fate. Here, a WD40 gene from *Raphanus sativus*, designated *TRANSPARENT TESTA GLABRA 1* (*RsTTG1*), was cloned and functionally characterized. Heterologous expression of *RsTTG1* in the *Arabidopsis thaliana* mutant *ttg1-22* background restored accumulation of anthocyanin and PA in the mutant and rescued trichome development. In radish, *RsTTG1* was abundantly expressed in all root and leaf tissues, independently of anthocyanin accumulation, while its MBW partners *RsMYB1* and *TRANSPARENT TESTA 8* (*RsTT8*) were expressed at higher levels in pigment-accumulating tissues. In yeast two-hybrid analysis, the full-length RsTTG1 protein interacted with RsTT8. Moreover, transient protoplast co-expression assays demonstrated that RsTTG1, which localized to both the cytoplasm and nucleus, moves from the cytoplasm to the nucleus in the presence of RsTT8. When co-expressed with *RsMYB1* and *RsTT8*, *RsTTG1* stably activated the promoters of the anthocyanin biosynthesis genes *CHALCONE SYNTHASE* (*RsCHS*) and *DIHYDROFLAVONOL 4-REDUCTASE* (*RsDFR*). Transient expression of *RsTTG1* in tobacco leaves exhibited an increase in anthocyanin accumulation due to activation of the expression of anthocyanin biosynthesis genes when simultaneously expressed with *RsMYB1* and *RsTT8*. These results indicate that RsTTG1 is a vital regulator of pigmentation and trichome development as a functional homolog of AtTTG1.

## 1. Introduction

Anthocyanins, flavonoid-derived metabolites, have multiple physiological roles in plants, including the attraction of pollinators, protection against damage from ultraviolet radiation, and involvement in the stress response under cold and drought conditions [1,2]. Additionally, anthocyanin accumulation results in aesthetically pleasing displays of various colors, in addition to providing humans with antioxidant health benefits when consumed [3,4]. The anthocyanin biosynthetic mechanisms are, therefore, an important topic for research related to basic plant physiology and to the improvement of human health.

The anthocyanin biosynthetic pathway has been well-characterized in many plant species [5], including the elucidation of the genes involved in its transcriptional regulation and the biosynthetic pathway. Transcription factors (TFs), including R2R3 MYB, basic helix–loop–helix (bHLH), and WD40 proteins, are known to interact with each other to form MYB–bHLH–WD40 (MBW) complexes that actively regulate anthocyanin biosynthesis [6,7]. The R2R3 MYB proteins possess conserved motifs known as two imperfect repeats (named R2 and R3) in their N terminus, which bind the DNA and their counterpart bHLH proteins. The C-terminal region of these MYB proteins is responsible for their transcription-regulatory activity. The bHLH proteins form transcriptional complexes by interacting with both the R2R3 MYB and WD40 proteins and directly regulate the expression of the anthocyanin biosynthesis genes. The WD40 proteins stabilize the interaction between the MYB and bHLH TFs via a docking platform, but lack transcriptional regulatory ability. In eudicots and monocots, some WD40 proteins have been functionally demonstrated to be indispensable for anthocyanin or PA biosynthesis, as well as for the determination of epidermal cell fate and formation, including ANTHOCYANIN 11 (PhAN11) from petunia (*Petunia × hybrida*) [8], TRANSPARENT TESTA GLABRA 1 (AtTTG1) from *Arabidopsis* (*Arabidopsis thaliana*) [9], WD REPEAT (PfWD) from perilla (*Perilla frutescens*) [10], MdTTG1 from apple (*Malus domestica*) [11], PALE ALEURONE COLOR 1 (ZmPAC1) from maize (*Zea mays*) [12], OsTTG1 from rice (*Oryza sativa*) [13], and FhTTG1 from freesia (*Freesia hybrida*) [14].

Radish (*Raphanus sativus* L.) is a member of the Brassicaceae family and is one of the most important taproot vegetable crops worldwide. The main edible component of radish is the taproot, which stores starch and other nutritious compounds, such as minerals and phytochemicals [15]. Radish taproots can be white, red, purple-pink, green, or even bicolor depending on the accumulation and distribution of anthocyanins and chlorophyll. Previous studies of radish reported that R2R3 MYB RsMYB1 and bHLH TF TRANSPARENT TESTA 8 (RsTT8) act as transcriptional regulators of anthocyanin biosynthesis, with their co-expression resulting in anthocyanin accumulation in heterologous tobacco (*Nicotiana tabacum*) plants [16,17,18]. A recent study showed that a frame-shift mutation of RsMYB1 results in the abnormal development of taproot skin color [18]. These results demonstrated that RsMYB1 and RsTT8 are key components of an MBW complex and are actively involved in anthocyanin biosynthesis. Despite these insights, the corresponding WD40 protein component of this MBW complex remains unknown. 

This study describes the identification and functional characterization of the radish WD40 protein, RsTTG1. *RsTTG1* is expressed in roots and leaves, regardless of anthocyanin accumulation. A yeast two-hybrid (Y2H) analysis revealed that the full-length RsTTG1 protein could directly interact with RsTT8, but not RsMYB1. Moreover, RsTTG1 can move from the cytoplasm to the nucleus in a manner either dependent on or synergic with RsTT8. A complementation analysis using the *Arabidopsis ttg1-22* mutant revealed that heterologously expressed *RsTTG1* restored anthocyanin and PA pigmentation as well as trichome production. A promoter activation assay and transient expression in tobacco leaves verified that *RsTTG1* likely enhances the transcription level of flavonoid biosynthesis genes as a member of the radish MBW complex. 

## 2. Results

### 2.1. Isolation of RsTTG1 cDNA and its Phylogenetic Analysis

**Figure 1:**
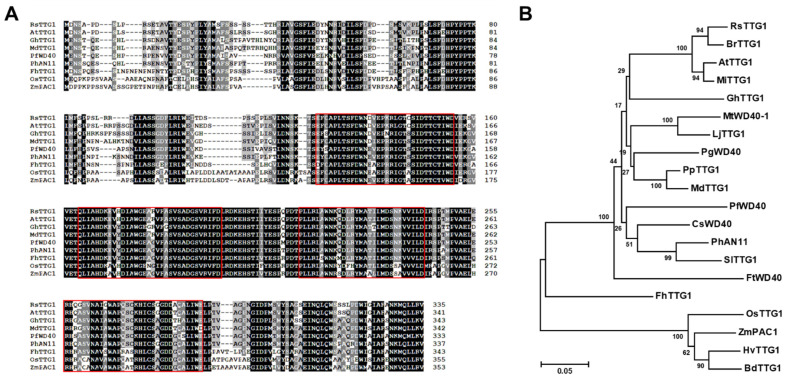
Phylogenetic relationships of RsTTG1 and selected WD40 proteins from other plant species. (**A**) Sequence alignment of RsTTG1 and its closest homologs, which are known to regulate flavonoid biosynthesis. Absolutely conserved residues are highlighted in black, while partially conserved residues are shown in gray. The positions of the WD repeats are shown in the red boxes. (**B**) Phylogenetic tree of RsTTG1 and related WD40 proteins from other plants. The phylogenetic tree was constructed using the neighbor-joining method with MEGA6 software. The GenBank accession numbers of the WD40 protein sequences are as follows: *Arabidopsis thaliana* AtTTG1 (Q9XGN1); *Brachypodium distachyon* BdTTG1 (XP_003570109); *Brassica rapa* BrTTG1 (ABQ10570); *Camellia sinensis* CsWD40 (QLI42590); *Fagopyrum tataricum* FtWD40 (APG16027); *Freesia hybrida* FhTTG1 (QJW70306); *Gossypium hirsutum* GhTTG1 (AAM95641); *Hordeum vulgare* HvTTG1 (XP_044952062); *Lotus japonicus* LjTTG1 (BAH28880); *Malus domestica* MdTTG1 (GU173813); *Matthiola incana* MiTTG1 (CAE53274); *Medicago truncatula* MtWD40-1 (ABW08112); *Oryza sativa* OsTTG1 (BAF09665); *Perilla frutescens* PfWD (BAB58883); *Petunia hybrida* PhAN11 (AAC18914); *Prunus persica* PpTTG1 (ACQ65867); *Punica granatum* PgWD40 (HQ199314); *Solanum lycopersicum* SlTTG1 (XP_004235332); and *Zea mays* ZmPAC1 (AAM76742).

To investigate the mechanism regulating anthocyanin biosynthesis in radish, WD40 genes isolated with leaves from the radish cultivars ‘948’ (white skin and white flesh; WSWF) and ‘Artesia’ (red skin and red flesh; RSRF) were cloned using degenerative PCR, 5′-RACE, and 3′-RACE. The isolated WD40 gene showed a 100% sequence identity between WSWF and RSRF and was subsequently named *RsTTG1*. *RsTTG1* has a 1,008 bp open reading frame (ORF) encoding a polypeptide of 335 amino acids. The deduced RsTTG1 protein displays the highly conserved characteristics of TTG1 homologs in other plant species (Figure 1A). Previous studies reported that these WD40 proteins have highly conserved WD40 motifs, which usually comprise a 40-amino acid tandem repeat characterized by Gly–His (GH) and Trp–Asp (WD) doublet residues [19]. The RsTTG1 protein shares the conserved characteristics of other WD40 proteins known to be involved in anthocyanin and PA biosynthesis in plants; the four WD40 repeats consisted of 30–33 amino acids and highly conserved two-amino acid sequences (WD, FD, LD, and WE) at the end of each WD-repeat motif [14,20,21,22,23].

In a phylogenetic tree of WD40 proteins involved in anthocyanin or PA biosynthesis, we found that RsTTG1 was grouped into the same cluster as WD40 proteins from *Arabidopsis thaliana* and *Brassica rapa*, consistent with their close taxonomic relationship with radish (Figure 1B). To identify the genomic structure of RsTTG1, we performed a PCR analysis using genomic DNA and cDNA (Supplementary Figure S1). The PCR fragments amplified from genomic DNA or cDNA showed almost identical lengths. A comparison of the genomic DNA and cDNA sequences revealed that RsTTG1 did not possess introns within its protein-coding region, which was consistent with other TTG1 homologs identified [21,24,25]. The radish RsTTG1 sequence was deposited into the GenBank database (accession number OP271757).

### 2.2. Anthocyanin Biosynthesis Genes Are Highly Expressed in Red Tissues

**Figure 2:**
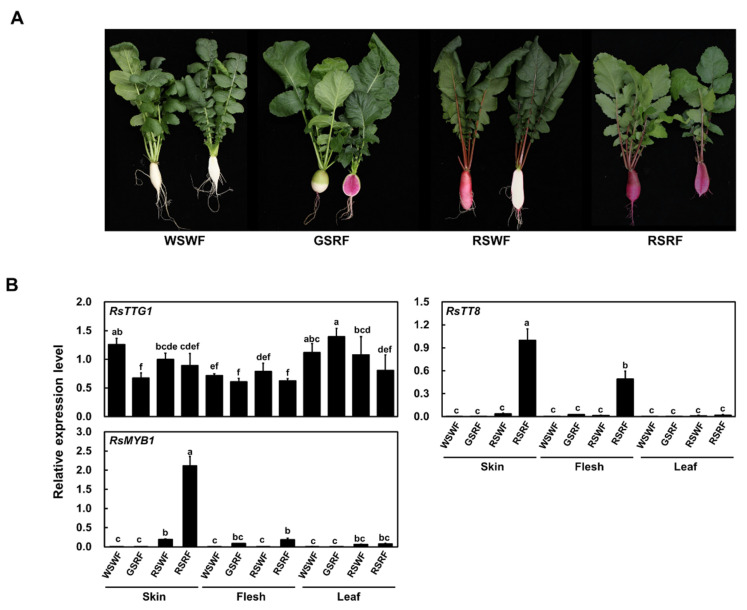

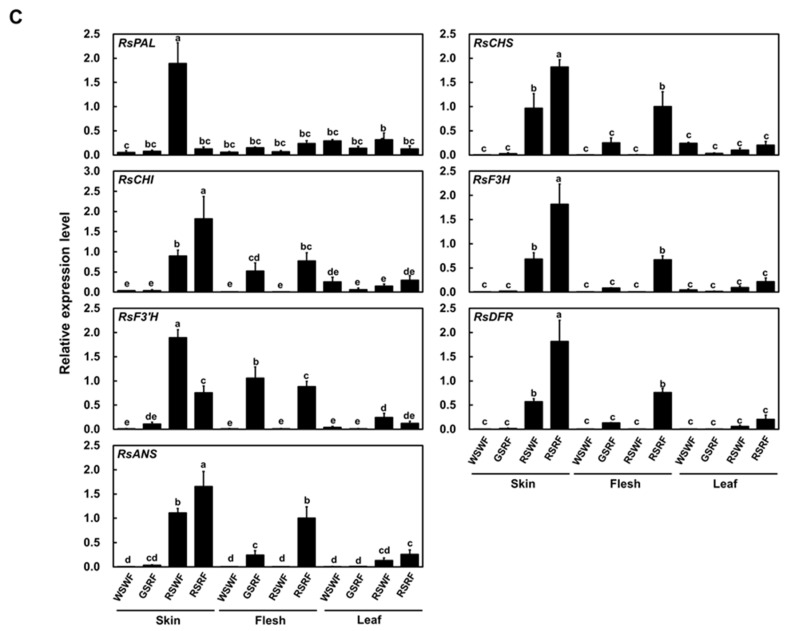
Expression of *RsTTG1* and other anthocyanin regulatory and biosynthesis genes in various radish cultivars with different pigmentation patterns. (**A**) Visual phenotypes of the four radish cultivars at the mature stage, with whole taproots on the left and sectioned taproots showing their flesh color on the right of each image. WSWF: white skin and white flesh; GSRF: green skin and red flesh; RSWF: red skin and white flesh; RSRF: red skin and red flesh. (**B**) Relative transcript levels of anthocyanin biosynthesis regulators. (**C**) Relative transcript levels of anthocyanin biosynthesis genes. Results are means ± SD from three independent biological replicates. *RsRPII* was used as the internal reference gene. Different letters indicate significantly different values (*p* < 0.05), as determined using a two-way ANOVA followed by Duncan’s multiple range tests.

To investigate whether the expression of *RsTTG1* coincided with anthocyanin accumulation in radish, we evaluated its transcription in different tissues, including the skin and flesh of roots and leaves (Figure 2). A real-time quantitative PCR (RT-qPCR) analysis revealed that *RsTTG1* was similarly expressed in all root and leaf tissues, independent of their visible pigment accumulation phenotype. *RsMYB1* and *RsTT8*, known to be activators of anthocyanin biosynthesis [16,17,18], were highly expressed in the red taproot skin and flesh (Figure 2B). Likewise, the genes encoding the anthocyanin pathway were generally expressed to much higher levels in the anthocyanin-accumulating tissues than non-anthocyanin-accumulating tissues (Figure 2C). The general phenylpropanoid biosynthesis gene *PHENYLALANINE AMMONIA-LYASE* (*RsPAL*) was highly expressed in the taproot skin of GSRF, whose taproots had green skin and red flesh, but was otherwise similarly expressed in the root flesh and leaves of all radish cultivars tested. The early biosynthesis genes, including *CHALCONE SYNTHASE* (*RsCHS*), *CHALCONE ISOMERASE* (*RsCHI*), *FLAVANONE 3-HYDROXYLASE* (*RsF3H*), and *FLAVONOID 3ʹ-HYDROXYLASE* (*RsF3′H*), were highly expressed in the pigmented radish cultivars. Similarly, the transcript levels of the late biosynthesis genes *DIHYDROFLAVONOL 4-REDUCTASE* (*RsDFR*) and *ANTHOCYANIDIN SYNTHASE* (*RsANS*) were high in the root skin and root flesh of the pigmented radish cultivars, consistent with the expression pattern of *RsMYB1* and *RsTT8*. Taken together, these results confirmed that the pigmentation phenotype reflected the expression levels of anthocyanin biosynthesis genes across the different cultivars and that this occurred in tissues co-expressing *RsMYB1* and *RsTT8* but was not affected by the expression level of *RsTTG1*.

### 2.3. RsTTG1 Functions in Anthocyanin and PA Biosynthesis and Trichome Development

**Figure 3:**
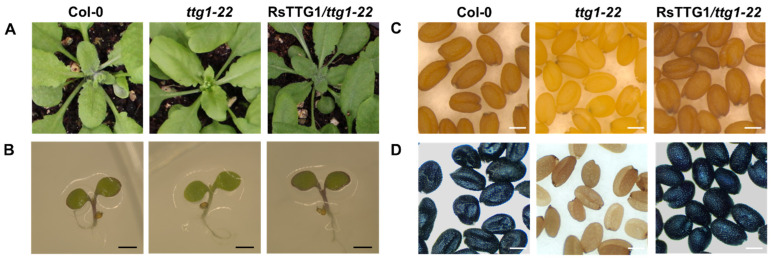
Complementation analysis of *RsTTG1* in the *Arabidopsis ttg1-22* mutant. Three-week-old seedlings (**A**), five-day-old seedlings (**B**), mature seeds (**C**), and DMACA-stained mature seeds (**D**) of wild type (Col-0), *ttg1-22* mutant, and T_3_ progeny of *ttg1-22* homozygotes transformed with *RsTTG1*. Black Bars, 1 mm. White Bars, 0.25 mm.

Heterologous expression of *RsTTG1* in the *Arabidopsis ttg1-22* mutant (CS330696) was used to test its regulatory role in the flavonoid biosynthesis pathway. The *Arabidopsis ttg1-22* mutant displays a glabrous (trichomeless) phenotype and does not accumulate anthocyanins in its cotyledons or hypocotyls. It also fails to accumulate PA in its seed coat, resulting in its yellow color (Figure 3). The *ttg1-22* mutant plants expressing *RsTTG1* displayed a rescued trichome development phenotype on the leaves, and they accumulated anthocyanin in their cotyledon and hypocotyl, visually resembling the wild type. The seeds of the *ttg1-22* plants expressing *RsTTG1* were brown, as observed in the wild type. To examine whether the rescued seed coat phenotype resulted from PA biosynthesis, the seeds were stained with 4-dimethylaminocinnamaldehyde (DMACA) and observed under a dissecting microscope. The DMACA staining revealed that the PA content in the mature transgenic *Arabidopsis* seeds was restored to the wild-type level. These results verified that RsTTG1 regulates multiple aspects of anthocyanin and PA biosynthesis and trichome development as a functional homolog of AtTTG1.

### 2.4. RsTTG1, RsTT8, and RsMYB1 Form a MBW Complex

**Figure 4:**
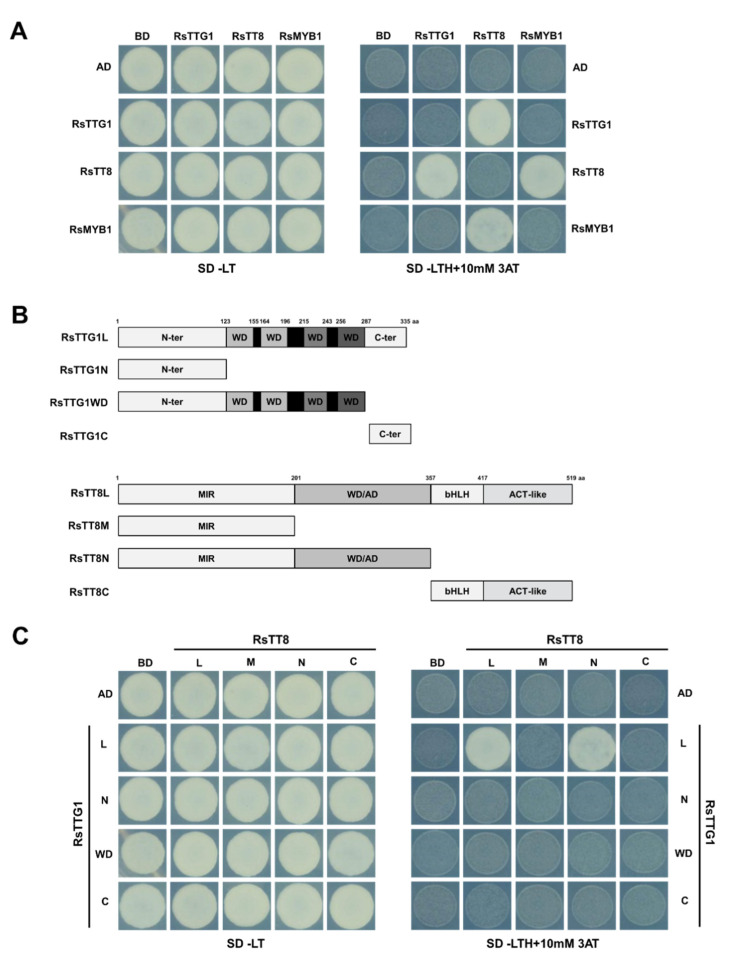
RsTTG1, RsTT8, and RsMYB1 form a ternary complex. (**A**) Interaction between RsTTG1, RsTT8, and RsMYB1 proteins, as revealed in a yeast two-hybrid (Y2H) analysis. (**B**) Schematic representation of constructs used to delineate the interaction interface between RsTTG1 and RsTT8. Amino acid positions are indicated in the diagrams. (**C**) Interactions between the truncated and full-length proteins of RsTTG1 and RsTT8. AD, activation domain; BD, binding domain; SD, synthetic defined medium; 3AT, 3-amino-1,2,4-triazole; SD/–TL, minimal medium lacking Trp and Leu; SD/–TLH + 3AT, synthetic defined medium lacking Trp, Leu, and His and containing 10 mM 3AT.

To investigate the role of RsTTG1 in the regulation of the flavonoid pathway, we performed Y2H assays with RsMYB1 and RsTT8, which are key regulators of flavonoid biosynthesis [16,17,18]. RsTTG1 physically interacted with RsTT8, but not RsMYB1 (Figure 4A). As was shown previously [17,18], RsMYB1 can interact with RsTT8. 

To define the interaction interface between RsTTG1 and RsTT8, we constructed Y2H constructs with full-length and partially truncated RsTTG1 and RsTT8 in-frame with the sequence encoding the GAL4 DNA-activation domain (AD) from the pGADT7 vector and the GAL4 DNA-binding domain (BD) from the pGBKT7 vector, respectively (Figure 4B). We co-transformed appropriate pairs of AD and BD constructs into the yeast strain MaV203 and tested their interaction on a stringent selective medium containing 10 mM 3-amino-1,2,4-triazole (3-AT), a competitive inhibitor of the yeast HIS3 enzyme. These assays showed that full-length RsTTG1 (RsTTG1L) can interact with partially truncated RsTT8N, as well as full-length RsTT8L, but cannot interact with partially truncated RsTT8M or RsTT8C, which do not contain the WD/AD region (Figure 4C). Notably, partially truncated RsTTG1 cannot interact with full-length or partially truncated RsTT8. These results indicate that the interaction between RsTTG1 and RsTT8 may be mediated by the full-length of RsTTG1 and the WD/AD region of RsTT8. Our findings confirmed that RsTT8 interacts with both RsMYB1 and RsTTG1, although RsMYB1 and RsTTG1 did not directly interact. We therefore hypothesize that RsTT8 serves as a bridge between RsMYB1 and RsTTG1, underlining its importance in the formation of the MBW complex.

### 2.5. Transport of RsTTG1 to the Nucleus Depends on its Interactions with RsTT8

**Figure 5:**
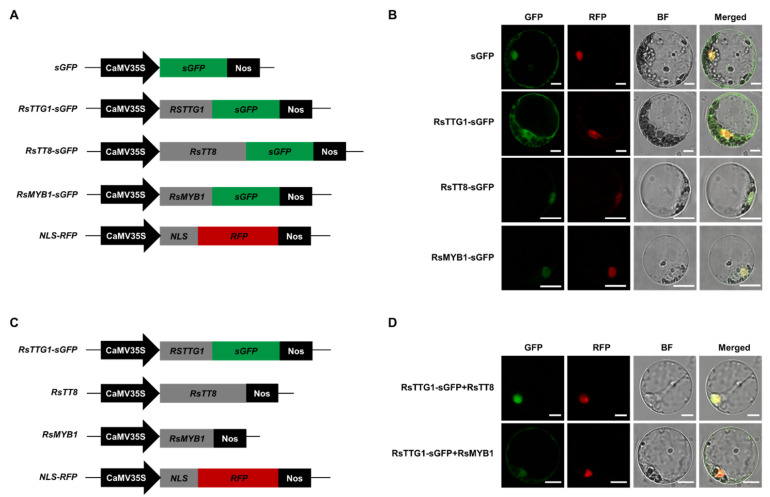
Proper intracellular localization of RsTTG1 is dependent on its interaction with RsTT8. (**A**) Schematic diagram of the constructs used in this experiment: *sGFP*, soluble GFP; *RsTTG1-GFP*; *RsTT8-GFP*; *RsMYB1-GFP*; and *NLS-RFP* (red fluorescent protein with nuclear localization sequence). (**B**) In vivo localization pattern of RsTTG1, RsTT8, and RsMYB1 in *Arabidopsis* protoplasts. Representative protoplasts accumulating each fusion protein are shown 16 h after transformation. Bars, 10 μm. (**C**) Schematic diagram of the constructs used in this experiment with different combinations of constructs. (**D**) In vivo intracellular localization pattern of RsTTG1, RsTT8, and RsMYB1 in *Arabidopsis* protoplasts. Representative protoplasts accumulating each fusion protein are shown 16 h after transformation. Bars, 10 μm.

To assess the subcellular distribution of RsTTG1, we fused this protein to green fluorescent protein (GFP) under the control of the cauliflower mosaic virus (CaMV) *35S* promoter (Figure 5A). We transiently transfected *Arabidopsis* mesophyll protoplasts with the 35S:RsTTG1-GFP construct, together with a nuclear localization marker consisting of red fluorescent protein (RFP) with the nuclear localization signal (NLS) of the SV40 large T antigen as a positive control (Figure 5B). An analysis of intracellular localization signals predicted that RsMYB1 and RsTT8 harbor a NLS and may, therefore, be localized in the nucleus, whereas RsTTG1 did not have a predicted NLS. Indeed, the fluorescence pattern of the 35S:RsTTG1-GFP construct showed a diffuse cellular distribution in both the nucleus and cytoplasm, similar to that of the control sGFP alone. Conversely, strong GFP fluorescence was detected in the nuclei of the protoplasts transfected with 35S:RsMYB1-GFP or 35S:RsTT8-GFP, as was expected.

To further investigate the movement of RsTTG1 into the nucleus for the formation of the MBW complex, we next analyzed the intracellular localization of 35S:RsTTG1-GFP in *Arabidopsis* mesophyll protoplasts co-transfected with either 35S:RsMYB1 or 35S:RsTT8 (Figure 5C). The green fluorescence signals were predominantly detected in the nucleus when 35S:RsTTG1-GFP and 35S:RsTT8 were co-expressed, while they were observed in both the nucleus and cytoplasm when 35S:RsTTG1-GFP was co-expressed with 35S:RsMYB1 (Figure 5D). This intracellular localization assay revealed that the nuclear import of RsTTG1 was dependent on its interaction partner RsTT8, but not RsMYB1. These results verified that RsTTG1 was able to participate in the conformation of the MBW complex due to its intracellular movement into the nucleus.

### 2.6. RsTTG1 Strengthens the Promoter Activity of RsCHS and RsDFR

Several studies reported that MBW complexes consisting of MYB, bHLH, and WD40 proteins collaboratively activated the transcription of anthocyanin or PA biosynthesis genes [6,26]. Generally, the WD40 protein acts as a scaffold protein onto which the complex is assembled and stabilizes the interaction between the MYB and bHLH TFs, rather than having a direct regulatory function [6,18,27]. Many studies indicated that WD40 proteins could only activate gene expression in the presence of other components of the MBW complex, such as bHLH proteins.

**Figure 6:**
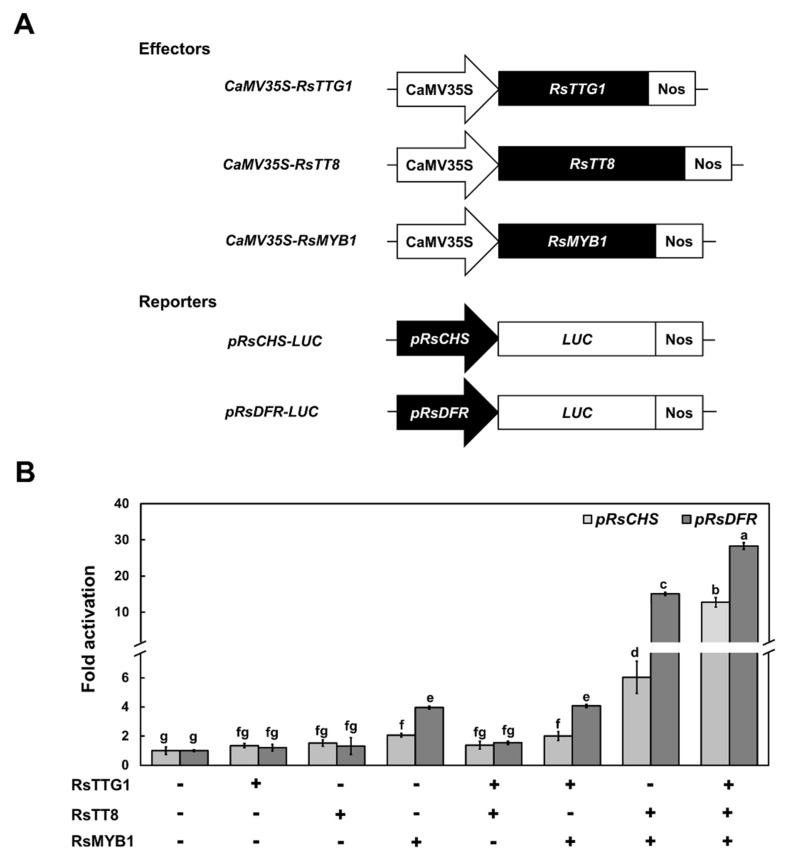
Transcriptional activation assay of the *RsCHS* and *RsDFR* promoters by RsTTG1, RsTT8, and RsMYB1. (**A**) Schematic diagrams of effector and reporter constructs used in the transcriptional activation assay. The effector construct harbors the coding sequence of RsTTG1, RsTT8, and RsMYB1, driven by the *35S* promoter and with the NOS terminator. The reporter constructs carry the *RsCHS* or *RsDFR* promoter driving *LUC* expression. (**B**) Regulatory consequences of the transient expression of RsTTG1, RsTT8, and/or RsMYB1 on *RsCHS* and *RsDFR* promoter activity. Results are mean values ± SD from three independent biological replicates. Different letters indicate significantly different values (*p* < 0.05), as determined using a one-way ANOVA followed by Duncan’s multiple range tests.

To further elucidate the roles of RsTTG1 in anthocyanin and PA biosynthesis, RsTTG1, RsTT8, and RsMYB1 were transfected into *Arabidopsis* protoplasts either alone or in different combinations to check their capacities in activating a luciferase (*LUC*) reporter gene driven by the *RsCHS* or *RsDFR* promoters (Figure 6). Similar to our previous study, the *RsCHS* and *RsDFR* promoters could be highly activated by the co-expression of RsMYB1 and RsTT8 [17,18]. By contrast, the expression of RsTTG1 alone or with RsTT8 failed to transactivate the *RsCHS* and *RsDFR* promoters. The co-expression of RsTTG1, RsTT8, and RsMYB1 significantly enhanced the promoter activity of *RsCHS* and *RsDFR* more than any other combination. Therefore, these data indicated that RsTTG1 could play a role in consolidating the activation efficiency of RsMYB1 and RsTT8 to promote the expression of anthocyanin biosynthesis genes such as *RsCHS* and *RsDFR*. 

### 2.7. RsTTG1 Enhances the Level of Flavonoid Biosynthesis Genes when Co-Expressed with RsMYB1 and RsTT8

**Figure 7:**
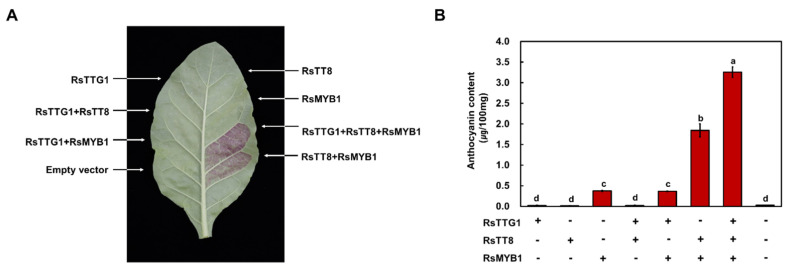
Functional analysis of RsTTG1, as a member of an MBW complex for anthocyanin biosynthesis. Visual phenotype and anthocyanin contents of tobacco leaves transiently infiltrated with *Agrobacterium* strains harboring an empty vector or constructs containing RsTTG1, RsTT8, and/or RsMYB1 in the indicated combinations. (**A**) Representative photograph of a transiently infiltrated tobacco leaf at 5 days after agroinfiltration. (**B**) Anthocyanin contents of the leaf sections shown in (**A**). Results are the means ± SD from three independent biological replicates. Different letters indicate significantly different values (*p* < 0.05), as determined using one-way ANOVA followed by Duncan’s multiple range tests.

Given that RsTTG1 has the ability to enhance the activation of *RsCHS* and *RsDFR* when acting together with RsMYB1 and RsTT8, we evaluated the function of RsTTG1 on anthocyanin biosynthesis via a transient assay in tobacco leaves. As expected, the transient expression of *RsTTG1* alone did not induce any visible pigment accumulation in tobacco leaves (Figure 7A). As shown in our previous study, the co-expression of *RsMYB1* and *RsTT8* could induce visible pigment accumulation and increased levels of anthocyanin in tobacco leaves (Figure 7B). Moreover, the expression of *RsTTG1* with *RsMYB1* and *RsTT8* resulted in tobacco leaves with a deep red color and higher anthocyanin contents than those expressing *RsMYB1* and *RsTT8* without *RsTTG1*, confirming that the participation of RsTTG1 in the MBW complex enhances anthocyanin accumulation.

**Figure 8:**
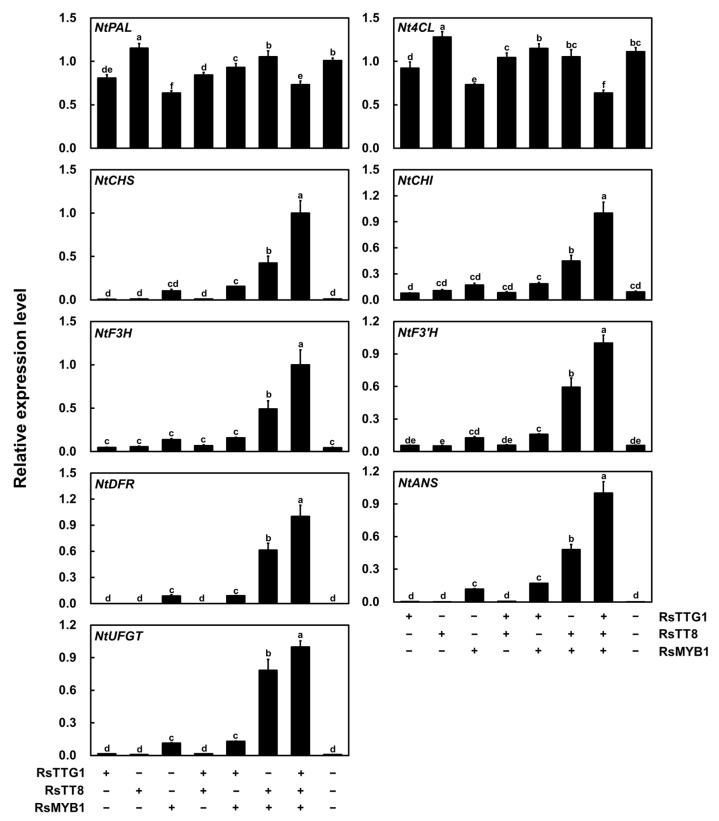
Relative transcription levels of the anthocyanin biosynthesis genes in tobacco leaves transiently expressing various combinations of the *RsTTG1*, *RsTT8*, and *RsMYB1* constructs. These transcription levels were determined using RT-qPCR, with *NtGAPDH* as the internal reference gene. Results are the means ± SD from three independent biological replicates. Different letters indicate significantly different values (*p* < 0.05), as determined using a one-way ANOVA followed by Duncan’s multiple range tests.

To explore the relationship between the transcription level of the anthocyanin biosynthesis genes and the anthocyanin contents, we measured the expression levels of nine structural genes involved in anthocyanin biosynthesis in infiltrated tobacco leaves (Figure 8). We focused on the upstream genes (*NtPAL* and *4-COUMARATE:COA LIGASE* (*Nt4CL*)), the early biosynthesis genes (*NtCHS*, *NtCHI*, *NtF3H*, and *NtF3′H*), and the late biosynthesis genes (*NtDFR*, *NtANS*, and *UDP-GLUCOSE: FLAVONOID 3-O-GLUCOSYLTRANSFERASE* (*NtUFGT*)). The transient expression of *RsTTG1* alone did not induce the transcription of any of the anthocyanin biosynthesis genes in the tobacco leaves, with the transcript levels remaining comparable to those of the control transfected with the empty vector and to those transiently expressing *RsTT8* alone. By contrast, the transient expression of *RsMYB1* alone and in combination with *RsTT8* activated the transcription of the anthocyanin biosynthesis genes, with the exception of *NtPAL* and *Nt4CL*, which showed high transcript levels in all samples, including the control transfected with the empty vector. Given that RsTTG1 interacts with RsTT8, we checked whether the expression of *RsTTG1* with either *RsTT8* or *RsMYB1* could activate the transcription of the anthocyanin biosynthesis genes. We concluded from these results that the combination of RsTTG1 and RsTT8 was not sufficient to induce the expression of the anthocyanin biosynthesis genes, despite their ability to bind to each other. Additionally, the result of co-expressing *RsTTG1* and *RsMYB1* was similar to that of the transient expression of *RsMYB1* alone. The co-expression of *RsTTG1*, *RsTT8*, and *RsMYB1* strongly activated the anthocyanin biosynthesis genes compared with that of *RsTT8* and *RsMYB1*. These results concluded that RsTTG1 is a crucial component of the MBW complex regulating anthocyanin biosynthesis. 

## 3. Discussion

### 3.1. RsTTG1 Restores the Phenotype of the Arabidopsis ttg1-22 Mutant 

The TTG1 protein is known to be a plant-specific regulator of multiple physiological processes, including anthocyanin and PA biosynthesis, epidermal cell fate determination, and seed coat mucilage production [28].

In this study, we isolated *RsTTG1*, which encodes a typical WD40 protein, using degenerative, 5′ RACE, and 3′ RACE analyses. Multiple alignments with other WD40 proteins showed that RsTTG1 has highly conserved characteristics, including four WD-repeat motifs that each harbor the amino acid residues WD, FD, LD, and WE present in other anthocyanin biosynthesis-related WD40 proteins from various species [11,12,13,14] (Figure 1). Our phylogenetic analysis showed that RsTTG1 is closely related to AtTTG1, which is known to be responsible for anthocyanin and PA accumulation and trichome development in *Arabidopsis* [9]. A complementation assay of RsTTG1 in the *Arabidopsis ttg1-22* mutant recovered its many deficient phenotypes, confirming that RsTTG1 is a functional homolog of AtTTG1 (Figure 3). Thus, it can be concluded that RsTTG1 could function in both anthocyanin and PA biosynthesis and trichome development.

### 3.2. Nuclear Transport of RsTTG1 Is Dependent on RsTT8, but Not RsMYB1

The import of TFs into the nucleus is important for their role in controlling gene expression. Our subcellular localization analysis in *Arabidopsis* mesophyll protoplasts demonstrated that RsMYB1 and RsTT8 were restricted to the nucleus, while RsTTG1 was distributed in both the nucleus and the cytoplasm (Figure 5B). Additionally, RsTTG1 was mobilized into the nucleus when in the presence of RsTT8, but not RsMYB1 (Figure 5D). These findings indicate that the nuclear import of RsTTG1 might depend on its interaction with the bHLH RsTT8, both of which could be components of an MBW complex.

Similar fine-tuning of TTG1 subcellular localization by TFs was previously reported in other plant species [10,14]. For instance, the transient expression of *FhTTG1* in freesia protoplasts revealed that the localization of FhTTG1 could be modified from a diffuse cellular distribution to a nuclear localization by co-expression with a bHLH TF gene, either *FhTT8L* or freesia *GLABRA3 (FhGL3L*) [14]. Similarly, the localization of PfWD could be changed from the cytoplasm to the nucleus in the presence of MYC-RP, a bHLH TF [10]. Additionally, the mobility of AtTTG1 is altered by the presence or absence of its binding partner bHLHs, such as AtGL3 [29]. These results emphasized that the interaction between bHLHs and WD40s was crucial for their cellular localization and the resulting transcriptional regulation of their downstream target genes.

### 3.3. RsTTG1, RsTT8, and RsMYB1 Interact to Form an Active MBW Complex

Previous studies reported that TTG1 proteins are involved in the regulation of multiple pleiotropic phenotypes, such as anthocyanin and PA accumulation, the patterning of root hairs and trichomes, and seed mucilage production [28]. For each process, TTG1 appears to participate via an MBW complex through its interaction with different MYB and bHLH proteins [30]. 

In the *Arabidopsis* *ttg1* mutant, the ectopic overexpression of genes encoding the MYB transcription factor AtPAP1 or the bHLH TF AtTT8 restores anthocyanin and PA biosynthesis in the leaves and seeds, implying that TTG1 is not crucial for the MYB–bHLH-induced expression of the anthocyanin and PA biosynthesis genes but may influence their level or activity [28]. Here, we revealed that the expression of *RsTTG1* can enhance the interaction activity between RsMYB1 and RsTT8, leading to the activation of their downstream target genes (Figure 7 and Figure 8); thus, the interaction between RsTTG1, RsTT8, and RsMYB1 is essential for these processes.

Previous studies reported that missense mutants of AtTTG1, *ttg1-23*, *ttg1-9*, and *ttg1-24*, possessing three independent amino acid substitutions at residues 197, 282, and 339, respectively, showed reduced anthocyanin levels in the seedling and PA levels in the seed coat, as well as diminished trichome production [31]. Similarly, the truncated mutant of AtTTG1, *ttg1-1*, in which the last 15 amino acids of the C terminus had been deleted, exhibited the typical phenotype of *ttg1* loss-of-function mutants [32]. Taken together, these findings suggest that the mutation of AtTTG1 at various positions along the protein resulted in a defective phenotype, both in terms of anthocyanin and PA accumulation and trichome development, indicating that many residues/regions of AtTTG1 are important for proper TTG1–bHLH interaction. Indeed, protein and protein interaction analyses verified that intact RsTTG1 was able to interact with its counterpart RsTT8, while the truncated RsTTG1 protein could not (Figure 4). The definition of interaction domains indicated that RsTTG1 interacted with the WD/AD region at the N terminus of RsTT8. These results reconfirmed the importance of the RsTT8 N-terminal region for its interaction with both RsMYB1 and RsTTG1 [17,18]. 

Using a transcriptional analysis of *Arabidopsis* protoplasts, we reaffirmed that activation of *RsCHS* or *RsDFR* promoters was controlled by RsMYB1 and enhanced by the presence of RsTT8 and RsTTG1 (Figure 6). A previous study reported that anthocyanin regulators MYB and bHLH TF targeted the cis-element of anthocyanin biosynthesis genes [33]. The promoter region of *RsCHS* and *RsDFR* genes had several bHLH-recognizing elements and MYB-recognizing elements with different spatial distances between cis-elements, respectively [17]. Promoter architecture has an important role in determining promoter activity [33]. Additionally, it demonstrated that RsTTG1 alone was not able to activate the promoters of *RsCHS* or *RsDFR*, but it could promote the full activation of these anthocyanin biosynthesis genes upon interacting with RsMYB1 and RsTT8. Based on these results, we concluded that RsTTG1 is a crucial component of the MBW complex regulating various genes associated with anthocyanin and PA biosynthesis.

Anthocyanin and PA are known to have important beneficial activities when consumed by humans, including the prevention of chronic diseases, certain cancers, and cardiovascular diseases, due to their strong antioxidant activities [3,4]. Deciphering the fundamental biosynthetic mechanism by which these metabolites are produced in radish taproots will provide the foundation for the manipulation of flavonoid metabolism, enabling the development of new radish cultivars with improved flavonoid contents to benefit human health. 

## 4. Materials and Methods

### 4.1. Plant Materials and Growth Conditions

Radish (*Raphanus sativus* L.) plants were grown in a greenhouse under short-day conditions at the National Institute of Agricultural Sciences (Jeonju, Korea). The following four radish cultivars with different taproot pigmentation patterns were used in this study: ‘948′ (from Asia Seed Co., Seoul, Korea) with white skin and white flesh (WSWF); ‘IT278184′ (from the National Agrobiodiversity Center, Jeonju, Korea) with green skin and red flesh (GSRF); ‘HongPi’ (from Nongwoo Seed Co., Suwon, Korea) with red skin and white flesh (RSWF); and ‘Artesia’ (Asia Seed Co.) with red skin and red flesh (RSRF). The taproots and leaves were harvested 50 days after sowing and immediately frozen in liquid nitrogen. The samples were stored at −80 °C before use.

The transformation experiments were conducted using the *Arabidopsis* mutant line *ttg1-22* (CS33096), which was obtained from the Arabidopsis Biological Resource Center. The *ttg1-22* plants were transformed with *RsTTG1* under the CaMV *35S* promoter following the floral-dip method. The transformed *Arabidopsis* plants were grown in soil under 16 h light/8 h dark conditions at 22 °C. Successfully transformed plants were selected by spraying with 0.3% Basta solution. 

Seeds of the Columbia (Col-0) ecotype, *ttg1-22* mutant, and homozygous T_3_ transgenic plants were surface-sterilized using sodium hypochlorite and germinated on 1/2 Murashige and Skoog (MS) medium [34] with 3% (*w*/*v*) sucrose. The plants were grown on MS medium or in soil under long-day conditions (16 h light/8 h dark, 100 μmol m^−2^ s^−1^) at 22 ± 2 °C. 

The transient expression assays were conducted using tobacco (*Nicotiana tabacum* cv. Xanthi) plants, which were grown in greenhouses under natural light at 26 ± 2 °C.

### 4.2. RNA Extraction, cDNA Synthesis, and Genomic DNA Isolation

To isolate the total RNA from the skin and flesh of roots, samples were first ground and treated with Fruit-mate for RNA Purification solution (Takara Bio, Kusatsu, Japan) to remove the polysaccharides and polyphenols. The samples were then treated with Plant RNA Purification Reagent (Thermo Fisher Scientific, Waltham, MA, USA) as described previously [18], before RNA was purified using the FavorPrep Plant Total RNA Mini Kit (Favorgen, Changzhi, Taiwan). For leaves of radish and tobacco plants, the total RNA was prepared from 100 mg samples with TRIzol reagent (Thermo Fisher Scientific) and purified using the FavorPrep Plant Total RNA Mini Kit (Favorgen), according to the manufacturer’s instructions. DNA contamination was removed by DNase I digestion (Ambion; Thermo Fisher Scientific). First-strand cDNA was synthesized from 2 μg of total RNA using amfiRivert cDNA Synthesis Platinum Master Mix (GenDEPOT, Barker, TX, USA).

Genomic DNA was extracted from 100 mg of radish leaves using the Plant Mini Kit (Qiagen, Hilden, Germany), according to the manufacturer’s instructions.

### 4.3. Total Anthocyanin Content Measurement and PA Detection

The total anthocyanin contents of the root skin, root flesh, and leaves were determined as previously described [35]. Briefly, 100 mg (fresh weight) samples were ground to powder in liquid nitrogen, then mixed with 600 μL extraction buffer (methanol containing 1% [*v*/*v*] HCl) and incubated for 6 h at 4 °C with moderate agitation. The addition of 200 μL water and 200 μL chloroform was followed by centrifugation to pellet the plant debris at 14,000 *g* for 5 min at 4 °C. The absorbance of the supernatant was recorded at 530 nm (A_530_) and 657 nm (A_657_) using a microplate reader, and the anthocyanin content was determined according to the formula A_530_ − (0.33 × A_657_). The experiments were performed with three independent biological replicates. 

To detect PA accumulation, mature seeds of Col-0, *ttg1-22*, and the T_3_ homozygous transgenic plants were harvested, stained with DMACA reagent (2% [*w*/*v*] DMACA in 3 M HCl/50% [*w*/*v*] methanol) for 1 week, and then washed three times in 70% (*v*/*v*) ethanol. 

### 4.4. RT-qPCR Analysis

Transcript levels were determined using RT-qPCR with the AccuPower 2x Greenstar qPCR Master Mix (Bioneer, Daejun, Korea) and the Bio-Rad CFX96 Detection System (Bio-Rad Laboratories, Hercules, CA, USA), according to the manufacturer’s instructions. The following RT-qPCR conditions were used: pre-denaturation at 95 °C for 5 min, with 40 cycles of denaturation at 95 °C for 15 s and annealing at 55 °C for 30 s. Gene expression was normalized to the internal reference genes *RNA POLYMERASE II* (*RsRPII*) and *GLYCERALDEHYDE 3-PHOSPHATE DEHYDROGENASE* (*NtGAPDH*) for radish and tobacco, respectively. The primers used for the RT-qPCR analysis are listed in Supplementary Table S1. Three independent biological replicates were used for each experiment.

### 4.5. Cloning and Sequence Analysis

The full-length ORF of *RsTTG1* was amplified from the cDNA and genomic DNA extracted from the ‘948′ (WSWF) and ‘Artesia’ (RSRF) cultivars using PCR with PrimeSTAR HS DNA Polymerase (Takara Bio) using the primer pair RsTTG1-F/R (Supplementary Table S2). The following PCR conditions were used: pre-denaturation at 98 °C for 5 min, followed by 35 cycles at 98 °C for 20 s, at 56 °C for 20 s, and 72 °C for 60 s, with a final extension at 72 °C for 5 min. All PCR amplicons were subcloned into the pENTR/D-TOPO vector (Thermo Fisher Scientific) for validation sequencing.

The nucleotide and deduced amino acid sequences of RsTTG1 were analyzed online. A structural analysis of the deduced proteins was carried out using the ExPASy Molecular Biology Server (http://cn.expasy.org/tools/, accessed on 5 October 2022). Multiple sequence alignments were generated using the CLUSTALW program (https://www.genome.jp/tools-bin/clustalw, accessed on 5 October 2022). A phylogenetic tree was constructed using the neighbor-joining method [36] in MEGA version 6 software [37]. Subcellular localization signals were predicted using the TargetP program (http://www.cbs.dtu.dk/services/TargetP/, accessed on 5 October 2022).

### 4.6. Y2H Assays

To examine the interaction between RsTTG1, RsTT8, and RsMYB1, their full-length ORFs were individually cloned into vectors pGADT7 and pGBKT7, which harbor GAL4-AD and the GAL4-BD, respectively, according to the manufacturer’s instructions. The constructs were introduced in pairs into the yeast strain MaV203. Yeast colonies were selected on synthetic defined (SD) medium lacking Trp and Leu and were replicated on SD medium lacking Trp, Leu, and His but containing 10 mM 3-AT, a competitive inhibitor of the yeast HIS3 enzyme. The plates were photographed after 2 days of incubation in the dark at 30 °C.

To identify the region of interaction between RsTTG1 and RsTT8, full and partial fragments of RsTTG1 were cloned into pGADT7, and full and partial fragments of the RsTT8 ORF were cloned into pGBKT7. The resulting constructs were introduced in pairs into the yeast strain MaV203. The transformants were plated on SD medium lacking Trp and Leu and were replica-plated onto SD medium lacking Trp, Leu, and His but containing 10 mM 3-AT. In all cases, the plates were photographed after 2 days in the dark at 30 °C.

### 4.7. Subcellular and Intracellular Localization Assays

The subcellular localizations of RsTTG1, RsTT8, and RsMYB1 were analyzed in *Arabidopsis* protoplasts, as described previously [38]. GFP fusion constructs were generated in the p326-sGFP plasmid, which contains the CaMV *35S* promoter. For the C-terminal GFP fusions, the ORFs of *RsTTG1*, *RsTT8*, and *RsMYB1* were individually amplified using gene-specific primer sets, which introduced an *Xba*I restriction site upstream of the ATG codon, using the InFusion Cloning System (Takara Bio). The resulting p326:RsTTG1-sGFP, p326:RsMYB1-sGFP, and p326:RsTT8-sGFP constructs were sequenced to confirm the absence of errors during PCR amplification. The plasmids were introduced into *Arabidopsis* mesophyll protoplasts using a polyethylene glycol (PEG)-mediated transformation. After incubation for 16–20 h in the dark at 25 °C, the cells were imaged using fluorescence confocal microscopy (Leica TCS SP8; Leica Microsystems, Wetzlar, Germany).

For the intracellular localization assays, the *RsMYB1* and *RsTT8* ORFs were inserted in the place of *sGFP* in the p326-sGFP plasmid, which was digested with *Xba*I and *Not*I, to generate 35S:RsMYB1 and 35S:RsTT8 constructs. *Arabidopsis* mesophyll protoplasts were co-transfected with p326:RsTTG1-sGFP and either 35S:RsMYB1 or 35S:RsTT8 constructs in the presence of PEG. After incubation for 16–20 h in the dark at 25 °C, the cells were imaged using fluorescence confocal microscopy. Signals of GFP fluorescence (493–546 nm) and RFP fluorescence (581–652 nm) were detected and imaged using a Leica TCS SP8 confocal microscope.

### 4.8. Promoter Activation Assay

For the transcriptional activity assays, the coding sequences of *RsTTG1*, *RsTT8*, and *RsMYB1* were inserted in place of *sGFP* in the p326-sGFP plasmid, which was digested with *Xba*I and *Not*I as described above, to generate effector constructs. To generate the reporter constructs, the promoter regions of *RsCHS* and *RsDFR* were individually amplified using PCR and then cloned into the p326-LUC vector, as previously described [18]. The reporter constructs also contained the *Renilla* *luciferase* (*REN*) gene driven by the *UBIQUITIN10* (*UBQ10*) promoter as an internal control. *Arabidopsis* mesophyll protoplasts were co-transfected with different combinations of reporter and effector constructs, as previously described [18]. The firefly and Renilla luciferase activity was measured using a dual-luciferase assay system (Promega, Madison, WI, USA), according to the manufacturer’s protocol. The normalized reporter activity was calculated from the LUC/REN ratio; this ratio was then set to 1 for transient transfection in the absence of the effector.

### 4.9. In Planta Assays of RsTTG1 Function

For the transient expression assays, the ORFs of *RsTTG1*, *RsMYB1*, and *RsTT8* were individually subcloned into the pENTR/D-TOPO vector (Thermo Fisher Scientific, Waltham,MA, USA) and incorporated into the Gateway destination vector pB2GW7 (VIB-Ghent University, Ghent, Belgium) using several Gateway cloning steps. The resulting constructs pB2GW7-RsTTG1, pB2GW7-RsMYB1, and pB2GW7-RsTT8 were individually introduced into *Agrobacterium* strain GV3101. For the transient assay, Agrobacteria harboring the genes of interest were inoculated onto the abaxial leaf surface of eight-week-old tobacco plants. Leaf color was monitored 5 days later, as described previously [18].

## 5. Conclusions

In this study, we characterized RsTTG1 as a member of the MBW complex, alongside its partners RsMYB1 and RsTT8, regulating anthocyanin and PA biosynthesis and trichome development in radish. A Y2H analysis and subcellular localization assay confirmed that RsTTG1 could interact with RsTT8, which was required for its intracellular movement. Through the transient expression assay using *Arabidopsis* protoplasts and tobacco infiltration, it was confirmed that the expression of *RsTTG1* can enhance anthocyanin accumulation in cooperation with *RsMYB1* and *RsTT8*. Taken together, these findings suggest that *RsTTG1* is a candidate target for the enhancement of anthocyanin and PA biosynthesis via metabolic engineering.

## Data Availability

Data supporting the findings of this study are available from the corresponding author, Sun-Hyung Lim, upon request.

## Supplementary Materials

The following supporting information can be downloaded at: https://www.mdpi.com/article/10.3390/ijms231911973/s1.
