# Supplementary material for: RsTTG1, a WD40 Protein, Interacts with the bHLH Transcription Factor RsTT8 to Regulate Anthocyanin and Proanthocyanidin Biosynthesis in Raphanus sativus
Source: Int J Mol Sci. 2022 Oct 9;23(19):11973. doi: 10.3390/ijms231911973 (PMC9570178; doi:10.3390/ijms231911973)
Supplement: Supplementary file 1 [file ijms-23-11973-s001.zip › Supplementary Table and Figure with revision 1005.pdf]

Supplementary Table S1. Gene IDs and specific primers for RT-qPCR analysis.

Gene name	Gene ID	Forward primer (5'-3')	Reverse primer (5'-3')
RsTTG1	Rs168100.1[a]	AACAGCAAGACGTCCGAGTT	GATGTCGTGGACCTCCTTGT
RsTT8	Rs527700.1[a]	AGTGATCGGAGCTGAGGAAA	ACTTGCTTCCTCCTCGCATA
RsMYB1	Rs388430.1[a]	GTGCATGGACTGCTGAAGAA	CAGTCCGACCGGGTAATCTA
RsPAL	Rs215330.1[a]	CGTCTCCTCAGTGGCTAG	CGTGAATCGCTTTGTTCCT
RsCHS	Rs065380.1[a]	GTGACTGGAACTCCCTCT	CTCTCATCTTCTCAGCCTTG
RsCHI	Rs260580.1[a]	TCCATCCTCTTCGCTCTC	GACACACGGTTCTTTCCAA
RsF3H	Rs341050.1[a]	TTACAAGCCACACGAGAC	ATGGTCGCCTAGATTAACAAC
RsF3'H	Rs392880.1[a]	AGACTCCATCCACCAACACC	TCTCGGGTCGAAACGATAAC
RsDFR	Rs305760.1[a]	GCTCATCAACGAAGGCTTTC	TTATTTAAAGCCATTTATAG
RsANS	Rs055270.1[a]	GAAGTTGGTGGCTTAGAAGAG	ATGTTGTGTAGAATCAAGGTCAA
RsRPII	Rs306620.1[a]	ATCACGCTAAATGGTCTCCT	GCTGCTCTCAATCAAGTCAATC
NtPAL	NM_001325423[b]	ATTGAGGTCATCCGTTCTGC	ACCGTGTAACGCCTTGTTTC
Nt4CL	NM_001325738[b]	TCATTGACGAGGATGACGAG	TGGGATGGTTGAGAAGAAGG
NtCHS	KU949020[b]	TTGTTCGAGCTTGTCTCTGC	AGCCCAGGAACATCTTTGAG
NtCHI	NM_001325287[b]	GTCAGGCCATTGAAAAGCTC	CTAATCGTCAATGCCCCAAC
NtF3H	NM_001325083[b]	CAAGGCATGTGTGGATATGG	TGTGTCGTTTCAGTCCAAGG
NtF3'H	XM_016592903[b]	AGGCTCAACACTTCTCGT	CATCAACTTTGGGCTTCT
NtDFR	NM_001325732[b]	AACCAACAGTCAGGGGAATG	TTGGACATCGACAGTTCCAG
NtANS	MF445066[b]	TGGCGTTGAAGCTCATACTG	GGAATTAGGCACACACTTTGC
NtUFGT	NM_001325312[b]	CAATGTTTGGGATGGTGTCA	TTCCTCCTCTGCCTCTTTCA
NtGAPDH	XR_001648122[b]	GGTGTCCACAGACTTCGTGG	GACTCCTCACAGCAGCACCA

[a] Radish genome database, http://radish-genome.org/; [b] NCBI, https://www.ncbi.nlm.nih.gov

Supplementary Table S2. List of primers used for cloning and vector construction in this study.

Usage	Primer name	Primer sequence
Gene cloning	5'race-TTG1-R1	CCCCCCAGGCGATGTCGTGGACCTCCTTGTC
	5'race-TTG1-R2	GATGGTGGAGTGCTCCTTGTCGCGGAG
	3'race-TTG1-F1	ACCGGACTCTCTACCTAGATCCGAAACCGCCG
	3'race-TTG1-F2	CGCCATGTCCTTCTCCTCCTCCTCCTCCAC
	RsTTG1-F	ATGGACAACTCAGCACCGGACTCTCTA
	RsTTG1-R	TCAAACTCTAAGGAGCTGCATTTTATTAGC
Yeast two hybrid	pGBKT7-RsTTG1-F	CATGGAGGCCGAATTCATGGACAACTCAGCACCG
	pGBKT7-RsTTG1-R	GGATCCCCGGGAATTCAACTCTAAGGAGCTGCATTT
	pGBKT7-RsTT8-F	CATGGAGGCCGAATTCATGGATGAATCAAGTATTATAC
	pGBKT7-RsTT8M-R	GGATCCCCGGGAATTCGAAGAAACTCTTCATGTGTTCAAC
	pGBKT7-RsTT8N-R	GGATCCCCGGGAATTCAGGAACTCTCAAGATCATGTGTTTG
	pGBKT7-RsTT8C-F	CATGGAGGCCGAATTCCATGAAGAAGACGAAGAAGTAG
	pGBKT7-RsTT8-R	GGATCCCCGGGAATTCGAGTTTATTTTGAGATAT
	pGBKT7-RsMYB1-F	CATGGAGGCCGAATTCATGGAGGGTTCGTCCAAAG
	pGBKT7-RsMYB1-R	GGATCCCCGGGAATTCCACAGTCTCTCCATCTAAC
	pGADT7-RsTTG1-F	GGAGGCCAGTGAATTCATGGACAACTCAGCACC
	pGADT7-RsTTG1N-R	CACCCGGGTGGAATTCGGACGTCTTGCTGTTGTT
	pGADT7-RsTTG1WD-R	CACCCGGGTGGAATTCCGTCGGCAACTCCCA
	pGADT7-RsTTG1C-F	GGAGGCCAGTGAATTCATGGACAACTCAGCACCG
	pGADT7-RsTTG1-R	CACCCGGGTGGAATTCAACTCTAAGGAGCTGCATTT
	pGADT7-RsTT8-F	GGAGGCCAGTGAATTCATGGATGAATCAAGTATTATAC
	pGADT7-RsTT8-R	CACCCGGGTGGAATTCGAGTTTATTTTGAGATAT
	pGADT7-RsMYB1-F	GGAGGCCAGTGAATTCATGGAGGGTTCGTCCAAAG
	pGADT7-RsMYB1-R	CACCCGGGTGGAATTCCACAGTCTCTCCATCTAAC
Subcellular/ Intraceullar localization	p326-RsTTG1-sGFP-F	CACGGGGGACTCTAGAATGGACAACTCAGCACCG
	p326-RsTTG1-sGFP-R	CCATGGATCCTCTAGAAACTCTAAGGAGCTGCATTT
	p326-RsTT8-sGFP-F	CACGGGGGACTCTAGAATGGATGAATCAAGTATTATAC
	p326-RsTT8-sGFP-R	CCATGGATCCTCTAGAGAGTTTATTTTGAGATATGATTT
	p326-RsMYB1-sGFP-F	CACGGGGGACTCTAGAATGGAGGGTTCGTCCAAAG
	p326-RsMYB1-sGFP-R	CCATGGATCCTCTAGACACAGTCTCTCCATCTAAC
	p326-RsTT8-F	CACGGGGGACTCTAGAGGATCCATGGATGAATCAAGTATTA
	p326-RsTT8-R	TTTGAACGATCGCGGCCGCCTAGAGTTTATTTTGAGATATG
	p326-RsMYB1-F	CACGGGGGACTCTAGAGGATCCATGGAGGGTTCGTCCAAA
	p326-RsMYB1-R	TTTGAACGATCGCGGCCGCTTACACAGTCTCTCCATCTAACA
In planta assay	RsTTG1-F	ATGGACAACTCAGCACCGGACTCTCTA
	RsTTG1-R	TCAAACTCTAAGGAGCTGCATTTTATTAGC
	RsTT8-F	ATGGATGAATCAAGTATTATACCGGTATGG
	RsTT8-R	CTAGAGTTTATTTTGAGATATGATTTGATGG
	RsMYB1-F	ATGGAGGGTTCGTCCAAAGGG CTGAG
	RsMYB1-R	TTACACAGTCTCTCCATCTAACAGGCT
	pDONR-RsTTG1-F	AAAAAAGCAGGCTCCATGGACAACTCAGCACCG
	pDONR-RsTTG1-R	GTACAAGAAAGCTGGGTCTCAAACTCTAAGGAGCTG

	pDONR-RsTT8-F	AAAAAAGCAGGCTCCATGGATGAATCAAGTATTA
	pDONR-RsTT8-R	GTACAAGAAAGCTGGGTCCTAGAGTTTATTTTGAGATATG
	pDONR-RsMYB1-F	AAAAAAGCAGGCTCCATGGAGGGTTCGTCCAAAGG
	pDONR-RsMYB1-R	GTACAAGAAAGCTGGGTCTTACACAGTCTCTCCATC
Promoter activation	pUC-proRsCHS-LUC-F	AGGCTCTAGAGGATCCGAACCCCACCTTAAAAACTTC
	pUC-proRsCHS-LUC-R	TTGGCGTCTTCCATGGATTAAACCAACTAGGTTTTCACTAG
	pUC-proRsDFR-LUC-F	AGGCTCTAGAGGATCCCATGATCTATTCAGAAAGTTGTTTT
	pUC-proRsDFR-LUC-R	TTGGCGTCTTCCATGGTTTTGTGTGTGTTGAAAAGATGGA

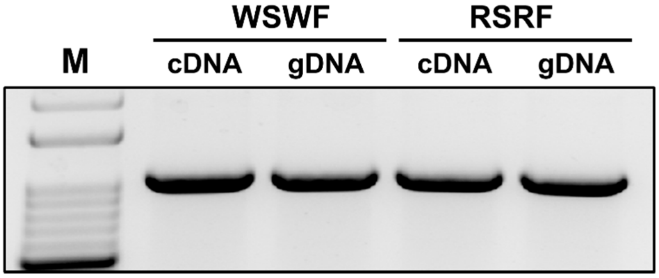

Supplementary Figure S1. Gel electrophoresis of amplified fragments of the RsTTG1 using radish cDNA or genomic DNA as template. The RsTTG1 sequences were amplified by specific primers using cDNA or genomic DNA of radish cultivar WSWF and RSRF, respectively. M: 100 bp DNA size marker.
